# P04 Urinary tract infections in HSCT: how big is the problem?

**DOI:** 10.1093/jacamr/dlad077.008

**Published:** 2023-08-02

**Authors:** Ioannis Baltas, Konstantinos Kavallieros, Ioannis Konstantinou, Malick M Gibani, Frances Davies, Jiri Pavlu

**Affiliations:** Department of Haematology, Imperial College Healthcare NHS Trust, London, UK; Faculty of Medicine, Imperial College London, London, UK; Faculty of Medicine, Imperial College London, London, UK; Department of Infectious Disease, Faculty of Medicine, Imperial College London, London, UK; Department of Infectious Disease, Faculty of Medicine, Imperial College London, London, UK; Department of Infectious Diseases, Imperial College London, London, UK; Department of Haematology, Imperial College Healthcare NHS Trust, London, UK

## Abstract

**Objectives:**

Urinary tract infections (UTIs) in HSCT recipients are understudied. We aimed to describe the incidence, clinical and laboratory findings of patients with UTIs during HSCT.

**Methods:**

This was a retrospective analysis of all patients admitted for HSCT between 1 January 2020 and 31 December 2022 in a large London tertiary centre. Patient-level data were collected from the electronic medical record.

**Results:**

There was a total of 400 admissions for HSCT during the study period, 56.25% (227/400) for autografts and 43.25% (173/400) for allografts. Median age was 55 years (IQR 43–62) and 59% of patients were male. A total of 588 infection episodes were recorded (mean 1.43 episodes per patient, 95% CI 1.39–1.56). Almost all patients (96.75%, 387/400) developed at least one infection episode during their inpatient stay for HSCT. The most common sources of infection were neutropenic fever (56.1%, 330/588) and non-neutropenic fever (17%, 100/588), followed by central line-associated bloodstream infections (10.2%, 60/588) and hospital- or ventilator-associated pneumonias (7%, 41/588). UTIs accounted for only 2% (12/588) of the total infection episodes. Only two UTIs (16.7%, 2/12) were associated with bacteraemia. A total of 1307 urine cultures were sent for all patients (3.23 per patient) and 93.5% of all patients had at least one urine culture. Growth of a specific pathogen was observed in 50/1307 cultures (3.8% positivity rate). The most common isolates included *Escherichia coli* (50%, 25/50) and *Enterococcus* spp. (22%, 11/50). The most common clinical syndrome for patients with positive cultures was neutropenic fever 40%, 20/50), followed by UTI (22%, 11/50) and non-neutropenic fever (6%, 3/50). 32% of positive samples (16/50) were considered to represent colonization. Pyuria was uncommon (8%, 4/50), including in patients with UTIs (18.2%, 2/11), as was haematuria (10%, 5/50 for all patients, 9.1%, 1/11 for patients with UTI).

**Conclusions:**

UTIs account for a small proportion of infections in HSCT recipients during their inpatient stay. Urine culture results are challenging to interpret, as they are often associated with undifferentiated fever syndromes or represent colonization. Urine microscopy results are unreliable in HSCT patients and should not be used to rule in or rule out UTI.

